# DEPDC5 deficiency contributes to resistance to leucine starvation via p62 accumulation in hepatocellular carcinoma

**DOI:** 10.1038/s41598-017-18323-9

**Published:** 2018-01-08

**Authors:** Yuki Mizuno, Shu Shimada, Yoshimitsu Akiyama, Shuichi Watanabe, Tomomi Aida, Kosuke Ogawa, Hiroaki Ono, Yusuke Mitsunori, Daisuke Ban, Atsushi Kudo, Shigeki Arii, Shoji Yamaoka, Minoru Tanabe, Shinji Tanaka

**Affiliations:** 1 Department of Molecular Oncology, Graduate School of Medicine, Tokyo Medical and Dental University, Tokyo, Japan; 2 Department of Hepato-Biliary-Pancreatic Surgery, Graduate School of Medicine, Tokyo Medical and Dental University, Tokyo, Japan; 3 Laboratory of Molecular Neuroscience, Medical Research Institute, Tokyo Medical and Dental University, Tokyo, Japan; 4 Department of Molecular Virology, Graduate School of Medicine, Tokyo Medical and Dental University, Tokyo, Japan

## Abstract

Decrease in blood concentration of branched-chain amino acids, especially leucine, is known to promote liver carcinogenesis in patients with chronic liver disease, but the mechanism is unclear. We herein established hepatocellular carcinoma (HCC) cells knocked out for DEPDC5 by using the CRISPR/Cas9 system, and elucidated that cell viability of the DEPDC5 knockout (DEPDC5-KO) cells was higher than that of the DEPDC5 wild-type (DEPDC5-WT) under leucine starvation. Considering that autophagy deficiency might be involved in acquired resistance to leucine deprivation, we observed reduction of LC3-II followed by accumulation of p62 in the DEPDC5-KO, which induced reactive oxygen species (ROS) tolerance. DEPDC5 overexpression suppressed cell proliferation and tumorigenicity in immunocompromised mice, and triggered p62 degradation with increased ROS susceptibility. In clinical specimens of HCC patients, decreased expression of DEPDC5 was positively correlated with p62 overexpression, and the progression-free (PFS) and overall survival (OS) were worse in the DEPDC5-negative cases than in the DEPDC5-positive. Moreover, multivariate analysis demonstrated DEPDC5 was an independent prognostic factor for both PFS and OS. Thus, DEPDC5 inactivation enhanced ROS resistance in HCC under the leucine-depleted conditions of chronic liver disease, contributing to poor patient outcome. It could be a potential target for cancer therapy with oxidative stress control.

## Introduction

Hepatocellular carcinoma (HCC) is a disease with poor prognosis and frequently complicated with chronic hepatic disease including viral and alcoholic hepatitis, non-alcoholic steatohepatitis and cirrhosis^1^. Such patients usually suffer from nutritional disturbances, especially decrease in branched-chain amino acids (BCAAs) which is known as an important risk factor of HCC^2^. Two prospective studies have recently reported that BCAAs administration could reduce the risk for HCC in patients with cirrhosis^3,4^ and that among BCAAs, blood concentration of leucine was inversely correlated with HCC onset^5^. These clinical data suggest leucine deficiency might contribute to hepatocarcinogenesis.

On the other hand, amino acid deprivation activates autophagy in the liver, and this mechanism exhibits tumor suppressor roles in various types of tissues including liver^6^. Autophagy-deficient mice developed HCC with accumulation of p62, a selective substrate of autophagy^7^, and p62 ablation attenuated the genesis of diethylnitrosamine-induced HCC in mice^8^. These contradictory data of the epidemiological and animal studies imply that HCC cells could survive by disrupting autophagic flux even under leucine starvation.

Since Sabatini and collaborators have currently elucidated that leucine deficiency inhibits mTORC1 activity through the modulation of the GATOR1 and 2 complexes and then induces autophagy pathway^9,10^, we highlighted DEPDC5, a component with GAP activity of the GATOR1 complex. DEPDC5 was identified as a gene responsible for familial focal epilepsy^11^, and whole genome sequencing of 102 pancreatic neuroendocrine tumors detected DEPDC5 inactivation caused by mutation and copy number alteration in half of them^12^. Although two papers have previously mentioned the involvement of DEPDC5 in hepatitis C virus (HCV)-related HCC^13,14^, the molecular mechanism and clinical significance remain obscure.

In this study, to clarify biological and molecular roles of DEDPC5 in HCC, we derived DEPDC5 knockout (DEPDC5-KO) subclones from human HCC cell lines, and examined the cellular response under leucine starvation. In addition, we performed immunohistochemical analysis of human HCC samples, and identified how DEPDC5 deficiency could contribute to the patient outcome.

## Results

### Establishment of the DEPDC5-knockout HCC cells

**Figure 1:**
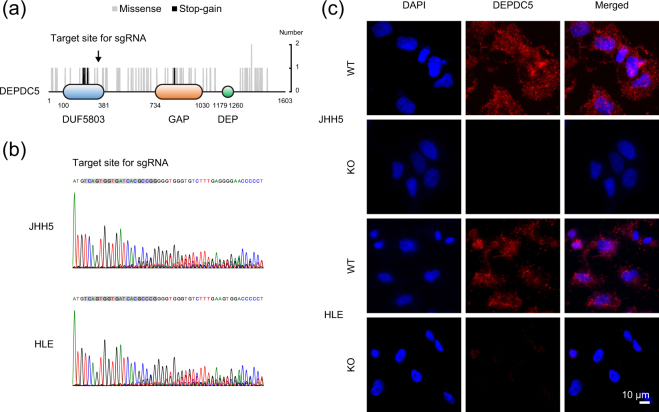
Establishment of the DEPDC5-KO HCC cells by using CRISPR/Cas9 system. (**a**) Schematics of the protein structure of DEPDC5. Grey and black bars show the position of amino acid substitutions induced by missense and stop-gain mutations in the ICGC public data. The arrow indicates the site that an sgRNA targets for knockout by using CRISPR/Cas9 technology in this study. (**b**) Sequence chromatograms of the DEPDC5-KO JHH5 and HLE cells around the sgRNA target site (grey background color). (**c**) Immunofluorescence analysis of the DEPDC5-WT and -KO JHH5 and HLE cells with DEPDC5 staining (red). Nuclei were counterstained with DAPI (blue). Magnification, ×200.

We first tried to establish the DEPDC5 knockout (DEPDC5-KO) subclones from human HCC cell lines by using CRISPR/Cas9 system. DEPDC5 contains three functional domains, DUF5803, GAP and DEP^15^. Among 85 mutations (missense 77; stop-gain 6; start-loss 1; start-gain 1) of DEPDC5 identified in HCC specimens registered on the ICGC Data Portal, stop-gain mutations were concentrated in the DUF5803 domain (Fig. 1a), which aids in binding to the other components of the GATOR1 complex. The mutation patterns of DEPDC5 was closely similar to those detected in individuals with familial focal epilepsy^16^. To examine DEPDC5 expression in HCC cells, we carried out immunocytochemical staining of the JHH5, HLE and HuH7 cells, which are cell lines isolated from HCC in patients with HCV infection. In the JHH5 and HLE cells, DEPDC5 appeared as a dot-like structure in the cytoplasm, whereas faint in the HuH7 (Supplementary Fig. 1). Thus, we prepared a single guide RNA (sgRNA) targeting the DUF5803 domain, and derived the DEPDC5-KO cells from the two DEPDC5-positive HCC cell lines, JHH5 and HLE. We also validated frameshift mutations (Fig. 1b) and no expression (Fig. 1c) of DEPDC5 by performing Sanger sequencing and immunocytochemistry in the transfomant pools, respectively.

### Cellular response of the DEPDC5-KO cells to leucine deprivation

**Figure 2:**
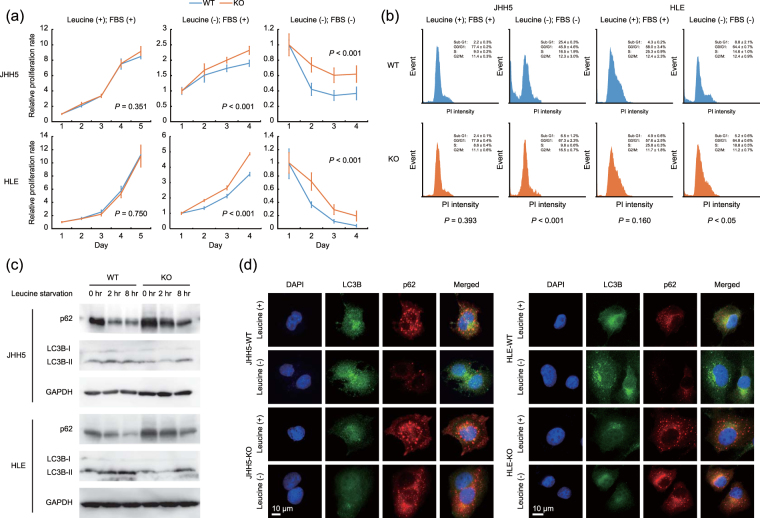
Cellular response of the DEPDC5-KO cells to leucine deprivation involved in autophagy pathway. (**a**) Proliferation curves of the DEPDC5-WT and -KO JHH5 and HLE cells. The value of each sample was relative to that at Day 1. Error bars are the mean ± S.D. *P* values were calculated by Welch’s *t*-test. (**b**) Flow cytometric analysis with PI staining. The percentage of each phase is the mean ± S.D. *P* values were calculated by Welch’s *t*-test. (**c**) Immunoblots of LC3B and p62. The cells were exposed to leucine-free medium for the indicated time periods. GAPDH was used as a loading control. (**d**) Immunofluorescence analysis of the DEPDC5-WT and -KO JHH5 and HLE cells under leucine-depleted conditions with LC3 (green) and p62 (red) staining. Nuclei were counterstained with DAPI (blue). Magnification, ×200.

To evaluate the biological effects of DEPDC5 disruption, we compared the proliferative ability of the DEPDC5-KO cells with that of the DEPDC5 wile-type (DEPDC5-WT). Although there was no difference under the standard conditions of growth medium including leucine and serum, the DEPDC5-KO JHH5 and HLE cells showed higher cell viability than the DEPDC5-WT when cultured in leucine-depleted medium with or without serum supplementation (Fig. 2a). Flow cytometric analysis with PI staining displayed the sub-G1 population increased to a lesser extent in the DEPDC5-KO HCC cells than in the DEPDC5-WT exposed to leucine- and serum-free medium, but not in complete medium (Fig. 2b), consistent with the cell proliferation assay described above. These findings suggested that DEPDC5 knockout could protect HCC cells from apoptotic events initiated by leucine starvation.

We next investigated which signaling pathways were activated or inactivated by knockout of DEPDC5 using microarray analysis. While Burza *et al*. have recently reported that DEPDC5 knockdown increases the expression levels of β-catenin and downstream genes of Wnt/β-catenin pathway in hepatic stellate cells^14^, the gene set enrichment analysis showed significant correlation between the expression of DEPDC5 and enhanced mTORC1 activity as well as anti-oxidative capacity, rather than Wnt/β-catenin pathway in the DEPDC5-KO HCC cells (Supplementary Fig. 2). Considering that mTOR-mediated autophagic flux could be abrogated in the DEPDC5-KO cells, we first monitored the expression levels of an autophagosomal marker LC3-II (Fig. 2c,d). After the DEPDC5-WT and -KO cells were exposed to leucine-free medium, LC3-II was rapidly induced in the DEPDC5-competent cells, but not in the DEPDC5-deficient, supporting the hypothesis above. Chloroquine increased LC3-II levels in the DEPDC5-competent JHH5 and HLE cells under leucine-free conditions, but not in the DEPDC5-deficient cells, indicating the inhibition of autophagy by DEPDC5 knockout (Supplementary Fig. 3). We next focused on p62, a key protein in autophagy-deficient HCC as a reactive oxygen species (ROS) scavenger^7,8^, and observed that p62 was gradually degraded in the DEPDC5-WT cells (Fig. 2c,d). In contrast, the expression levels of p62 were notably high even under the standard culture conditions and moderately decreased by leucine withdrawal in the DEPDC5-KO.

### Acquired resistance to oxidative stress in the DEPDC5-KO HCC cells through accumulation of p62

**Figure 3:**
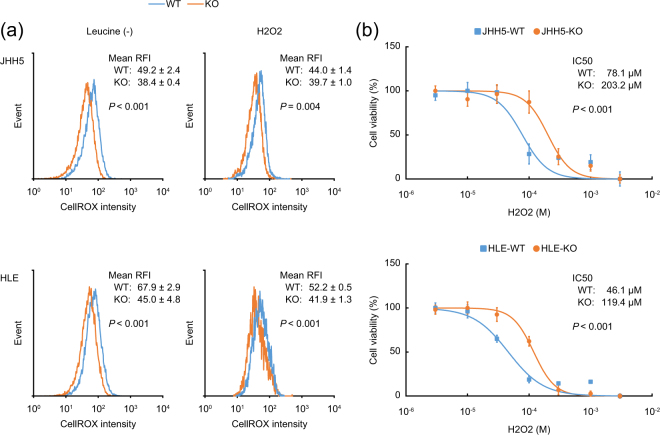
Reduction of cellular ROS levels in the DEPDC5-KO HCC cells. (**a**) Representative histogram images of cells with CellROX. The concentration of H2O2 was 100 μM in the right panels. The value of each mean relative fluorescence intensity (RFI) is the mean ± S.D. *P* values were calculated by Welch’s *t*-test. (**b**) Dose-response curves of the cell viability after H2O2 treatment. *P* values were calculated from the ANOVA table.

We also examined whether accumulation of p62 could show anti-oxidative effects in the DEPDC5-KO cells. By using CellROX Reagent, a fluorogenic probe for measuring oxidative stress, cellular ROS levels were significantly lower in the DEPDC5-KO than in the DEPDC5-WT under leucine-depleted conditions (Fig. 3a, left). Additionally, ROS generation by hydrogen peroxide (H2O2) treatment was inhibited in the DEPDC5-KO (Fig. 3a, right), resulting in strikingly decreased vulnerability to H2O2 (Fig. 3b). These data demonstrated that the DEPDC5-KO cells could showed enhanced tolerance to oxidative stress.

### Tumor suppressor roles of DEPDC5 ***in vitro*** and ***in vivo***

**Figure 4:**
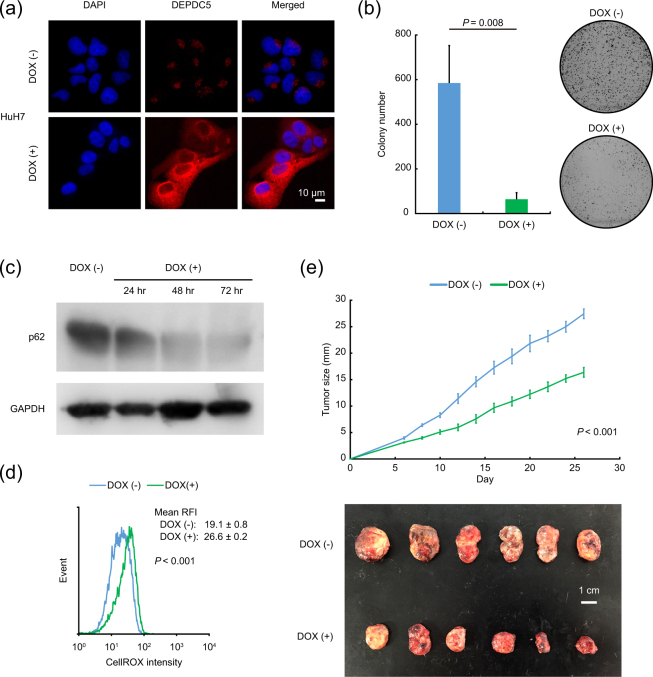
Inhibition of cancer cell growth with elevated cellular ROS levels by DEPDC5 overexpression. (**a**) Immunofluorescence analysis of the doxycycline (DOX)-inducible DEPDC5-expressing HuH7 cells with DEPDC5 staining (red). Nuclei were counterstained with DAPI (blue). Magnification, ×200. (**b**) Quantification of colony-forming efficiency. Error bars are the mean ± S.D. *P* values were calculated by Welch’s *t*-test. (**c**) Immunoblots of p62. The cells were exposed to medium containing doxycycline for the indicated time periods. GAPDH was used as a loading control. (**d**) Representative histogram images of cells with CellROX. The value of each mean RFI is the mean ± S.D. *P* values were calculated by Welch’s *t*-test. (**e**) *In vivo* tumorigenicity of doxycycline-inducible DEPDC5-expressing HCC cells. The upper and lower panels show growth curves of transplanted tumors and representative photo images. Error bars are the mean ± S.E in the upper panel. *P* values were calculated by Welch’s *t*-test. The white scale bar is 1 cm in the lower panel.

We also constructed sublines for doxycycline-inducible expression of DEPDC5 from the DEPDC5-negative HCC cells of HuH7. As expected, DEPDC5 overexpression triggered by doxycycline treatment was confirmed by immunocytochemical staining (Fig. 4a), and strongly repressed colony-forming capacity (Fig. 4b). In the DEPDC5-expressing HuH7 cells supplemented with doxycycline, p62 was gradually diminished (Fig. 4c) with an increase of ROS production over time (Fig. 4d), consistent with the abovementioned data of DEPDC5 knockout (Figs 2, 3). After subcutaneously injecting the inducible DEPDC5-expressing HCC cells into NOD/SCID mice, we fed them with or without doxycycline in drinking water, and identified delayed tumor growth in the mice administered by doxycycline (Fig. 4e). Overall, DEPDC5 could exhibit tumor suppressor roles in HCC by degrading p62 protein and then elevating cellular ROS levels.

### Clinical significance of DEPDC5 and p62 in HCC

**Figure 5:**
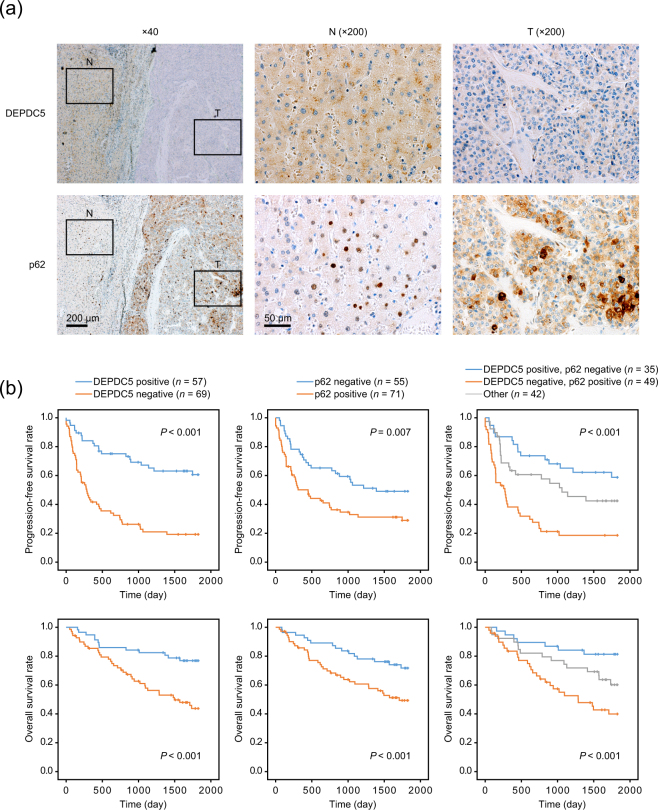
Relationship among DEPDC5 and p62 expression in HCC samples and patient prognosis. (**a**) Immunohistochemical analysis of DEPDC5 and p62 in a representative tissue sample including adjacent liver tissue (N) and cancer (T). Nuclei were counterstained with hematoxylin. In adjacent liver tissues of almost all cases, DEPDC5 was positive while p62 was negative. (**b**) Kaplan-Meier curves of the progression-free and overall survival in groups of HCC patients classified according to DEPDC5 and p62 expression patterns. *P* values were calculated by the log-rank test.

Immunohistochemical analysis of DEPDC5 was performed in 126 clinical specimens of human HCC, and then clinical significance of DEPDC5 expression was assessed. A dot-like staining of DEPDC5 resided in the cytoplasm (Fig. 5a), which resembled the staining patterns in immunocytochemistry as shown in Supplementary Fig. 1. While DEPDC5 expression was observed in the adjacent liver tissue of most cases, the expression levels of DEPDC5 were downregulated in 54.8% of HCC samples. The DEPDC5-negative cases had worse prognosis for progression-free (PFS) and overall survival (OS) than the DEPDC5-positive (Fig. 5b).

As documented by the previous paper^8^, p62 was highly expressed in the tumor area (Fig. 5a), and served as a potent indicator for poor outcome of HCC patients (Fig. 5b). We compared the staining patterns of DEPDC5 and p62, and revealed the inverse relationship between them (*P* < 0.001, Fisher’s exact test), consistent with the findings from the cell experiments (Fig. 2c,d). Furthermore, we divided all cases into three groups, the DEPDC5-negative and p62-positive, DEPDC5-positive and p62-negative, and other cases. In both PFS and OS, the DEPDC5-negative and p62-positive group showed the worst prognosis whereas the DEPDC5-positive and p62-negative did the best (Fig. 5b).

Next, there was significant difference in clinicopathological factors of blood concentration of AFP and PIVKA-II, size of tumor, and grade of portal vein infiltration between the DEPDC5-negative and -positive groups (Table 1). Univariate analysis suggested that several factors including DEPDC5 and p62 expression contributed to PFS and OS as displayed in Table 2. In multivariate analysis, only portal vein invasion (*P* = 0.013, *P* = 0.042) and DEPDC5 expression (*P* < 0.001, *P* = 0.013) were negatively associated with PFS and OS (Table 2), indicating that the downregulated expression levels of DEPDC5 could be an independent predictive marker for patient prognosis.

**Table 1:** Relationship between DEPDC 5 expression and clinicopathological factors.

Clinicopathological factor	DEPDC5 expression		*p* value
	Positive	Negative	
	(*n* = 57)	(*n* = 69)	
Gender, n male:female	51:6	52:17	0.062
Age, years mean ± SD	66.8 ± 9.2	64.1 ± 9.3	0.221
Liver function			
Platelet, 10^4^/µl, mean ± SD	14.0 ± 9.3	15.5 ± 7.3	0.186
Prothrombin time, %, mean ± SD	86.1 ± 14.8	85.5 ± 14.3	0.866
Albumin, mg/dl, mean ± SD	4.10 ± 0.40	4.00 ± 0.47	0.900
AST, U/L mean ± SD	39.0 ± 27.6	45.0 ± 49.4	0.232
ALT, U/L mean ± SD	34.0 ± 38.2	36.0 ± 52.1	0.750
Total bilirubin, mg/dl, mean ± SD	0.70 ± 0.36	0.80 ± 0.30	0.950
ICG-R15, %, mean ± SD	15.8 ± 8.7	14.8 ± 10.2	0.583
Background liver disease			
B:C:NBNC, n	11:18:18	15:27:11	0.448
Background liver pathology			
NL:CH:LC, n	4:33:20	8:25:36	0.052
Oral administration of BCAAs, n	2:55	8:61	0.088
Tumor factor			
AFP, ng/ml median (min.-max.)	7.6 (1.8–12287.0)	27.6 (2.3–398063.0)	<0.001
PIVKA-II, mAU/ml median (min.-max.)	79 (1–247360)	199 (15–132000)	0.044
Tumor size, cm, mean ± SD	3.8 ± 3.62	5.5 ± 3.06	0.004
Tumor number, n			
Solitary:multiple	41:16	45:24	0.448
Portal vein invasion, n			
Vp1:Vp > 2	52:5	49:20	0.006
Histological grade, n			
well:moderate:poor	20:26:11	13:39:17	0.118
p62 positive:negative, n	22:35	49:20	<0.001

AST aspartate aminotransferase, ALT alanine aminotransferase, ICG-R15 indocyanine green retention rate at 15 min, B hepatitis B, C hepatitis C, NBNC non-B non-C, NL normal liver, CH chronic hepatitis, LC liver cirrhosis, BCAAs branched chain amino acids, AFP α-fetoprotein, PIVKA-II protein induced by vitamin K absence or antagonist-II.

**Table 2:** Univariate and multivariate analysis of factors contributing to progression-free and overall survival.

Clinicopathological factor	Progression-free survival		Overall survival	
	Univariate analysis	Multivariate analysis	Univariate analysis	Multivariate analysis
	*p* value	*p* value	*p* value	*p* value
Gender, male versus female	1.000		0.410	
Age, years	0.639		0.754	
Liver function				
Platelet, 10^4^/µl	0.954		0.182	
Prothrombin time, %	0.075		0.035	0.115
Albumin, mg/dl	0.004	0.070	0.010	0.101
AST, U/L	0.006	0.255	0.023	0.275
ALT, U/L	0.113		0.165	
Total bilirubin, mg/dl	0.445		0.642	
Background liver disease				
Viral versus non-viral, n	0.010	0.066	0.038	0.229
C versus non-C, n	0.048	0.308	0.042	0.230
Background pathology				
LC versus non-LC, n	0.273		0.184	
Oral administration of BCAAs, n	0.600		0.518	
Tumor factor				
AFP, ng/ml	<0.001	0.035	<0.001	0.082
PIVKA-II, mAU/ml	0.030	0.737	0.006	0.153
Tumor size, cm	0.056		0.047	0.681
Tumor number, solitary versus multiple, n	0.137		0.451	
Histological grade				
well and moderate versus poor, n	0.016	0.370	0.012	0.202
Portal vein invasion, Vp1 versus Vp ≥ 2, n	<0.001	0.001	<0.001	0.036
p62 negative versus positive, n	0.008	0.590	0.012	0.104
DEPDC5 positive versus negative, n	<0.001	<0.001	<0.001	0.005

AST aspartate aminotransferase, ALT alanine aminotransferase, C hepatitis C, LC liver cirrhosis, BCAAs branched chain amino acids, AFP α-fetoprotein, PIVKA-II protein induced by vitamin K absence or antagonist-II.

## Discussion

Heiden *et al*. demonstrated that cancer proliferation depends on amino acids, rather than glucose^17^, and significance of amino acids in cancer development has attracted great attention in recent years. Dependency of amino acids is different for each type of malignancy through various molecular mechanisms^18–21^. Leucine deprivation causes apoptosis in melanoma since it fails to appropriately trigger autophagy^22^. Restriction of dietary serine and glycine can reduce tumor growth in intestinal cancer and lymphoma mouse models, and antagonizing the anti-oxidant response can reinforce the anti-tumor effects^23^. In HCC, decreased leucine blood concentration was positively correlated with carcinogenesis among blood metabolic factors^5^, suggesting some protective mechanisms against leucine deficiency. We herein highlighted a newly identified leucine sensor DEPDC5, and elucidated that DEPDC5 knockout rescued apoptotic cell death of HCC induced by leucine depletion whereas DEPDC5 overexpression restrained cell growth *in vitro* and *in vivo*, resulting that DEPDC5 could act as a potential tumor suppressor.

The reasons for DEPDC5 deficiency in liver cancer are as follows; the public data of The Cancer Genome Atlas (TCGA) project identified mutations of DEPDC5 in eight cases (2.1%; missense 7; stop-gain 1) of HCC specimens and worse overall survival of patients with DEPDC5 mutation (Supplementary Fig. 4a). Genome-wide analysis detected homozygous deletion in astrocytoma^24^, inactivation due to copy number alteration in pancreatic neuroendocrine tumor^12^, and single nucleotide variant in DEPDC5 involved in liver carcinogenesis or liver fibrosis, which is still controversial^13,14^. The mRNA expression level of DEPDC5 was downregulated in HCC samples and correlated with poor patient prognosis in the TCGA public data (Supplementary Fig. 4b). DEPDC5 contains a ubiquitination site (Lys 1543) as shown in Fig. 1a and then may be controlled by post-transcriptional mechanisms.

This study showed the increased aggregation of p62 in the DEPDC5-KO HCC cells and the inverse correlation between p62 and DEPDC5 expression in clinical specimens of HCC. Although as an autophagy adaptor at first, p62 has currently been identified as an oncogenic protein playing critical roles in cellular detoxification of oxidative stress via several pathways such as stabilization of NRF2 by antagonizing KEAP1^25^. The anti-oxidant response associated with p62 can contribute to tumor progression in the liver as addressed by two significant researches; one is that p62 is induced by carcinogen treatment in mice and promotes its mitogenic activity^8^, and the other is that mutations of NRF2 and KEAP1 account for approximately 6% and 4% of cases in HCC, respectively^26^.

Taken together, we examined each step of the cascade from leucine deprivation to poor prognosis of patients with HCC, and elucidated that the DEPDC5-KO HCC cells could acquire anti-oxidant ability through p62 accumulation and survive under leucine starvation, and that downregulated DEPDC5 expression was an independent predictive factor for patient outcome. Various drugs with direct or indirect effects on ROS metabolism have been available for cancer therapies^27^. A more detailed investigation of the roles of DEPDC5 deficiency in cancer cell metabolism will help to define better-targeted therapies.

## Methods

### Cell culture, animal studies, and human subjects

The human embryonic cell line (HEK293T) and human HCC cell lines (JHH5, HLE, and HuH7) were purchased from the American Type Culture Collection (Manassas, VA) or the Human Science Research Resources Bank (Osaka, Japan). They were cultured in DMEM or RPMI 1640 (Wako, Osaka, Japan) supplemented with 10% fetal bovine serum, 100 U/ml penicillin, and 100 μg/ml streptomycin (Invitrogen, Carlsbad, CA), maintained in a humidified incubator at 37 °C in 5% CO_2_, and harvested with 0.05% trypsin-0.03% EDTA (Invitrogen). For investigation of cellular response to leucine starvation, custom medium purchased from Biological Industries (Beit-Haemek, Israel) was used. Autophagy-flux assay by treatment with chloroquine was performed following the protocol^28^. NOD/SCID (NOD.CB17-*Prkdc* ^*scid*^/J) mice were purchased from Charles River Laboratories (Wilmington, MA). All mouse procedures were approved by the Institutional Animal Care and Use Committee of Tokyo Medical and Dental University (permission No. 0160074 A), and conducted under the guidelines established by it. A total of 126 patients who underwent curative hepatic resection for HCC at Tokyo Medical and Dental University Hospital between 2007 and 2010. With approval of the ethics committees of the Faculty of Medicine in Tokyo Medical and Dental University (permission No. 1080), written informed consent was obtained from all patients. Patients were anonymously coded in accordance with ethical guidelines, as instructed by the Declaration of Helsinki.

### Establishment of sublines with knockout of DEPDC5 from human HCC cell lines

The CRISPR-targeting sequence (5′-GTCAGTGGTGATCACGCCCGGGG-3′) used in this study was designed on the basis of the Opimized CRISPR Design web tool (http://crispr.mit.edu:8079/) for knockout of DEPDC5^29^. Oligos were cloned into the lentiCRISPR v2 vector (Addgene, plasmid #52961) generously provided form Dr. Feng Chang following the protocol^30^. HEK293T cells were transfected with the lentiviral vector, pCMVΔR8.2 and pHCMV-VSV-G (kind gifts from Dr. Irvin Chen) by using X-tremeGENE HP DNA Transfection Reagent (Sigma-Aldrich St. Louis, MO). Culture supernatants were collected and passed through 0.45 μm-membrane filters (Millipore, Billerica, MA) 60 hours after transfection. The JHH5 and HLE cells were infected for 12 hours in the supernatant containing 10 μg/ml polybrene (Nacalai Tesque, Kyoto, Japan), and then treated with 3 μg/ml puromycin (Invitrogen) two days after infection. Transformant pools were confirmed by DNA sequencing and immunofluorescence.

### Immunofluorescence

Cells were seeded onto small coverslips in 6-well plates and incubated at 37 °C for 24 hours to allow cell attachment. The cells were fixed with 4% paraformaldehyde at 4 °C for 15 minutes, permeabilized with 0.1% Triton X-100 for five minutes followed by incubation in 3% bovine serum albumin for 30 minutes at room temperature. The blocking buffer was removed, and the cells were incubated with primary antibodies for an hour at 4 °C. After washed with PBS, they were additionally incubated with fluorescence-conjugated secondary antibodies for an hour, and the cellular DNA was subsequently counterstained with Hoechst 33342 solution (Thermo Fisher Scientific, Rockford, IL). The slides were viewed with a fluorescent microscope (Carl Zeiss, Oberkochen, Germany). Primary antibodis against DEPDC5 (1:100, HPA055619; Sigma-Aldrich), LC3B (1:200, #3868; Cell Signaling Technology, Danvers, MA) and p62 (1:100, ab56416; Abcam, Cambridge, UK) were used.

### Cell proliferation assay

Cells were plated at a density of 2 × 10^3^ cells per well in 96-well plates under each culture conditions, and counterstained for nuclei with Hoechst 33342 solution (Dojindo, Kumamoto, Japan), and the number was estimated with IN Cell Analyzer 2000 (GE Healthcare, Buckinghamshire, UK).

### Cell cycle analysis

Cells were plated in 6-cm dishes and grown overnight. Twenty-four hours after growth medium was changed to the leucine-competent or -deficient, the cells were harvested, washed with PBS and fixed with 70% ethanol overnight at −20 °C. After rinsed with PBS containing 3% bovine serum albumin, the cells were resuspended in PBS with 50 μg/ml propidium-iodide solution (Sigma-Aldrich) and 10 μg/ml RNase A (Sigma-Aldrich) for 30 minutes on ice. The stained cells were counted by a FACSCalibur flow cytometer (BD Biosciences, San Jose, CA).

### RNA extraction and microarray analysis

Total RNA was extracted from cells by using RNeasy Protect Mini Kit (QIAGEN, Hilden, Germany). The integrity of the obtained RNA was confirmed by using 2100 Bioanalyzer (Agilent Technologies, Santa Clara, CA). Contaminating DNA was removed by digestion with RNase-Free DNase Set (QIAGEN). Complementary RNA was prepared from 100 ng of total RNA from each sample with 3′ IVT Express Kit (Affymetrix, Santa Clara, CA). The hybridization and signal detection of GeneChip Human Genome U133 Plus 2.0 Array (Affymetrix) were performed in accordance with the manufacturer’s instructions. The microarray datasets of a pair of the HLE-WT and -KO were normalized by using the robust multiarray average method in the R statistical software (version 3.0.3) and the Affy Bioconductor package. To investigate how the biological functions changed by DEPDC5 knockout during the acquisition of resistance to anti-angiogenic therapy, the gene set enrichment analysis (GSEA) was performed with the MSigDB gene sets (H: hallmark gene sets; version 5.2).

### Western blotting

Western blotting for total protein extracted from cells was performed as described previously^31^. Primary antibodies against GAPDH (1:1000, #5174; Cell Signaling Technology), LC3B (1:1000, #3868; Cell Signaling Technology) and p62 (1:200, ab56416; Abcam) were used.

### Detection of reactive oxygen species

Cells were seeded in 6-cm dishes a day before the assay. After incubated with each medium condition, the cells were additionally incubated in 5 μM CellROX Orange Reagent (Invitrogen) for an hour at 37 °C, washed with PBS, and then recovered with trypsin treatment. The fluorescence intensity produced by the CellROX Orange Reagent was determined with FACSCalibur. The mean relative fluorescence intensity (RFI) was calculated by using WinMDI 2.8 software.

### Establishment of sublines with inducible expression of DEPDC5 from human HCC cell lines

Overlap PCR technology was used to generate a lentiviral vector expressing C-terminal enhanced GFP (eGFP)-tagged DEPDC5. The entire coding sequences of DEPDC5 and eGFP were amplified by using the primer pair sets, 5′-ATGAATCGATATGAGAACAACAAAGGTCTACAAAC-3′ (forward) and 5′-CTCCTCGCCCTTGCTCACCATCGGGGCACTGGCATGCATC-3′ (reverse) for DEPDC5, and 5′-GATGCATGCCAGTGCCCCGATGGTGAGCAAGGGCGAGGAG-3′ (forward) and 5′-TATAGCGGCCGCTTACTTGTACAGCTCGTC-3′ (reverse) for eGFP from eGFP Depdc5 pLJM1 vector (Addgene, plasmid #46380; a kind gift from Dr. David Sabatini). The chimeric PCR product was digested with ClaI and NotI, and cloned into the same restriction enzyme cleavage site of the pRetroX-TRE3G plasmid (Clontech, Palo Alto, CA). The vector construct was termed as pRetroX-TRE3G-DEPDC5. A retroviral vector inducibly expressing eGFP (pRetroX-TRE3G-eGFP) was also constructed by using the primer pair sets, 5′-AGTTATCGATGCCACCATGGTGAGCAAGG-3′ (forward) and 5′-TATAGCGGCCGCTTACTTGTACAGCTCGTC-3′ (reverse) as control. Retrovirus packaging was performed by using Plat-E cells according to the similar protocol of lentivirus packaging described above. The HuH7 cells were sequentially infected with retroviruses produced from the pRetroX-Tet3G (Clontech) and pRetroX-TRE3G-DEPDC5 or pRetroX-TRE3G-eGFP plasmids and selected by 5 mg/ml G418 (Invitrogen) and 3 μg/ml puromycin, respectively. Transformant pools were used for subsequent analysis. For inducible expression of DEPDC5, the cells were cultured in growth medium including 1 mg/ml doxycycline (Sigma-Aldrich).

### Colony formation assay

Cells were plated at a density of 1 × 10^3^ cells per well in 6-cm dishes and incubated at 37 °C with medium containing doxycycline or not. There were no significant differences in colony-forming capacity of HuH7 without pRetroX-TRE3G-DEPDC5 under normal medium with and without DOX. The cells were fixed by 100% methanol, and counterstained for nuclei with crystal violet solution (Wako) fourteen days later. The number of the stained cells was estimated by using ImageJ 1.51 software.

### Tumor seeding

After suspended in 100 μl Matrigel (BD Biosceiences), 1 × 10^7^ cells were subcutaneously injected into NOD/SCID mice fed with or without doxycycline in drinking water (2 mg/ml). The size of the growing tumors was monitored every three days. There were no differences in appearances including food intake or body weight between mice fed with and without DOX administration (data not shown).

### Immunohistochemistry

Human liver tissue samples were sectioned (4 μm thick), and stained with an automated immunostainer (DISCOVERY XT; Ventana Medical Systems, Tucson, AZ) by using heat-induced epitope retrieval and a standard diaminobenzidine detection kit. Primary antibodies were the anti-DEPDC5 (1:200) and anti-SQSTM1/p62 (1:100). Secondary antibodies included donkey anti-goat IgG-B (1:200; sc-2042; Santa Cruz Biotechnology, Santa Cruz, CA) and universal secondary antibody (Ventana Medical Systems). All tissue sections were counterstained with hematoxylin. Immunoreactivity of more than 10% of carcinoma cells was assessed as positive for DEPDC5 and p62 independently by three investigators in a blind manner without knowledge of clinicopathologic data of patients.

### Statistical analysis

The mutation data of DEPDC5 in HCC were directly presented on the ICGC Data Portal (https://dcc.icgc.org/). The TCGA data for mutations and mRNA expression levels were downloaded from the cBioPortal site (http://www.cbioportal.org/). Statistical analysis was performed by using SPSS statistics Version 20.0 (IBM, Armonk, NY). The two-sided Welch’s *t* test was used to analyze for differences between continuous values of two independent groups. The χ^2^ test or Fisher’s exact test was applied to analyze categorical variables. Survival curves were constructed by using the Kaplan-Meier method and compared with the log-rank test. After univariate analysis, the significant variables were subjected to multivariate analysis by using the Cox proportional-hazards model. *P* values less than 0.05 were considered statistically significant.

## Electronic supplementary material

Supplementary Information
