# Supplementary material for: DEPDC5 deficiency contributes to resistance to leucine starvation via p62 accumulation in hepatocellular carcinoma
Source: Sci Rep. 2018 Jan 8;8:106. doi: 10.1038/s41598-017-18323-9 (PMC5758822; doi:10.1038/s41598-017-18323-9)

DEPDC5 deficiency contributes to resistance to leucine starvation via p62 accumulation in hepatocellular carcinoma

Yuki Mizuno[1,2], Shu Shimada[1], Yoshimitsu Akiyama[1], Shuichi Watanabe[1,2], Tomomi Aida[3], Kosuke Ogawa[2], Hiroaki Ono[2], Yusuke Mitsunori[2], Daisuke Ban[2], Atsushi Kudo[2], Shigeki Arii[2], Shoji Yamaoka[4], Minoru Tanabe[2], & Shinji Tanaka[1,2,*]

[1]Department of Molecular Oncology, Graduate School of Medicine, Tokyo Medical and Dental University, Tokyo, Japan. [2]Department of Hepato-Biliary-Pancreatic Surgery, Graduate School of Medicine, Tokyo Medical and Dental University, Tokyo, Japan. [3]Laboratory of Molecular Neuroscience, Medical Research Institute, Tokyo Medical and Dental University. [4]Department of Molecular Virology, Graduate School of Medicine, Tokyo Medical and Dental University.

Supplementary Figure Legends

Supplementary Fig. 1. Immunocytochemical analysis of hepatoma cell lines (JHH5, HLE, and HuH7) with DAPI counter staining. Magnification, ×200.

Supplementary Fig. 2. Enrichment plots of gene sets associated with the DEPDC5-KO HLE cells. NES, normalized enrichment score. FDR, false discovery rate.

Supplementary Fig. 3. Autophagy-flux assay by treatment with chloroquine. The cells were exposed to leucine-free medium with or without chloroquine (50 μM) for the indicated time periods. GAPDH was used as a loading control.

Supplementary Fig. 4. Prognosis of hepatocarcinoma cases registered in ICGC public data. (**a**) Kaplan-Meier curves of overall survival in groups of patients with HCC harboring or not DEPDC5 mutation. (**b**) Kaplan-Meier curves of overall survival in groups of HCC patients divided by mRNA expression levels of DEPDC5. Patients harboring HCC with the top and bottom quartile expression of DEPDC5 were classified into the DEPDC5 high and low.

Supplementary Figure 1

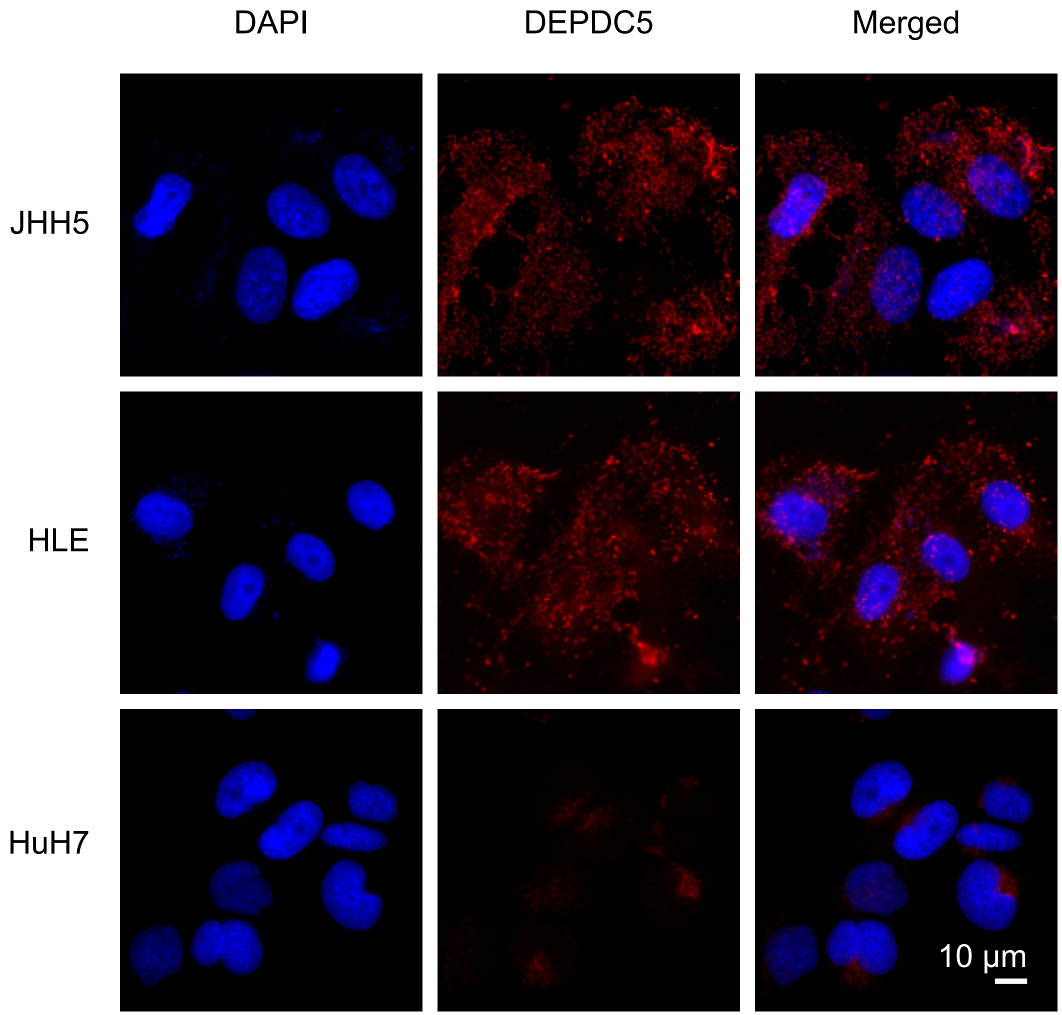

Supplementary Figure 2

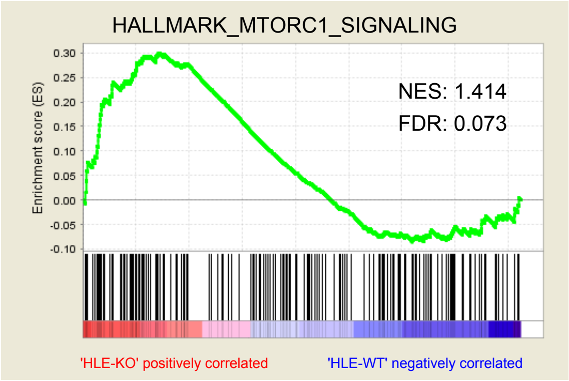

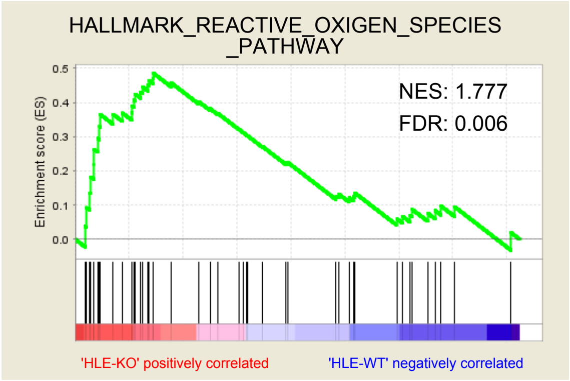

Supplementary Figure 3

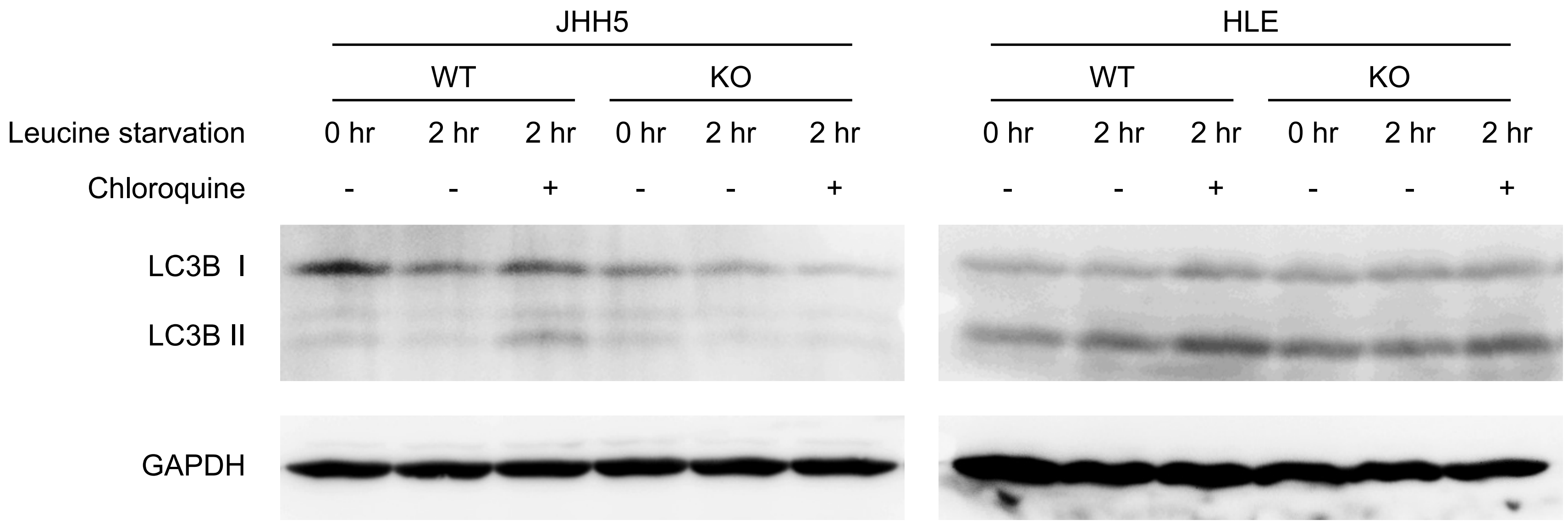

Supplementary Figure 4

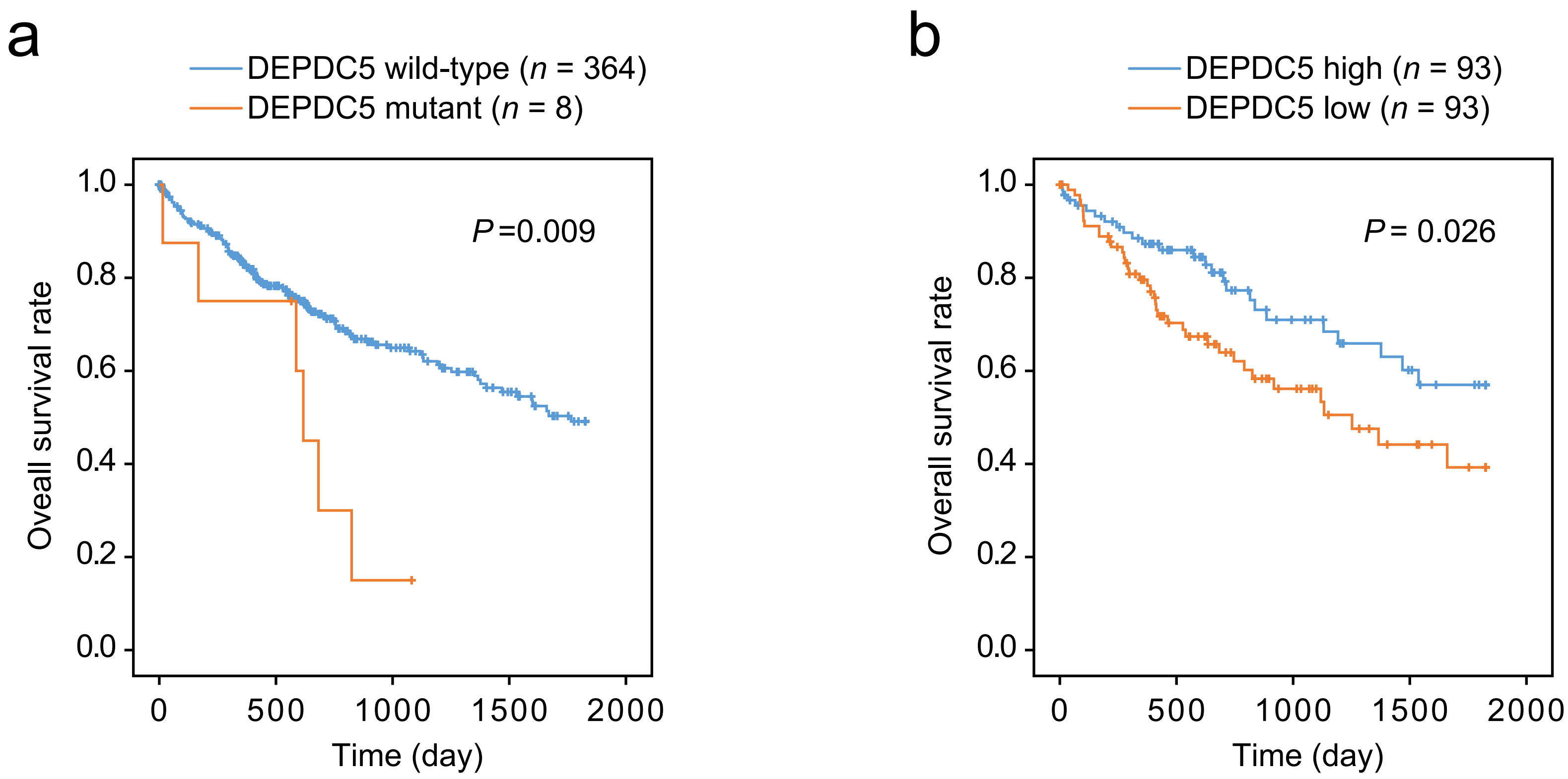

Original images of blots

Figure 2c. JHH5 - p62

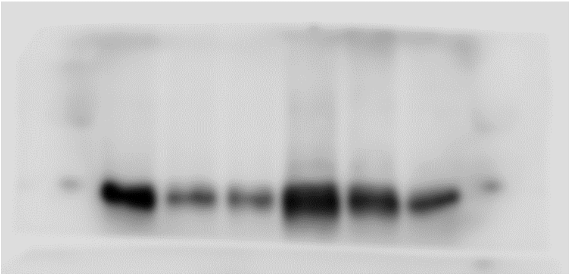

Figure 2c. HLE - p62

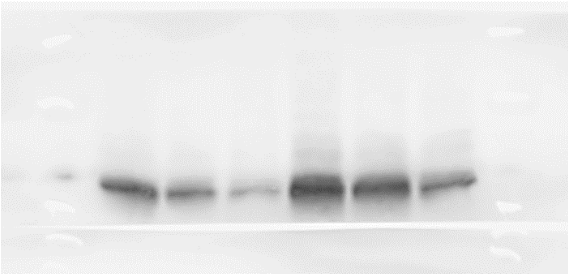

Figure 2c. JHH5 - LC3B

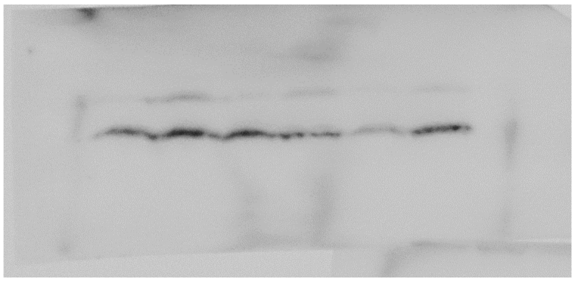

Figure 2c. HLE - LC3B

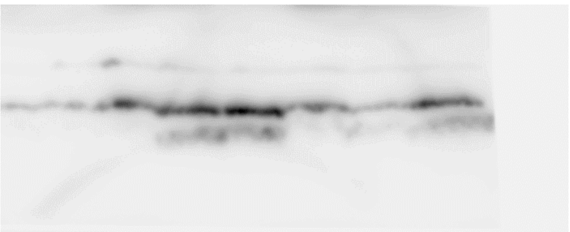

Figure 2c. JHH5 - GAPDH

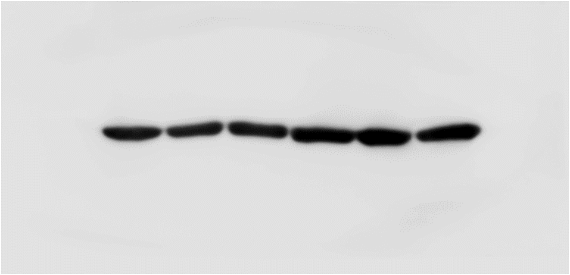

Figure 2c. HLE - GAPDH

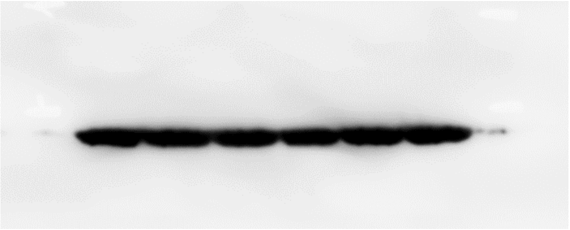

Figure 4c. HuH7 - p62

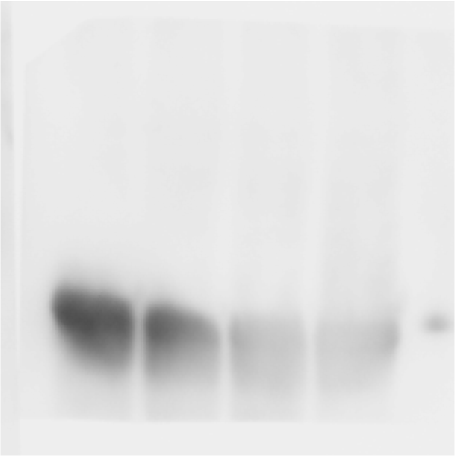

Figure 4c. HuH7 - GAPDH

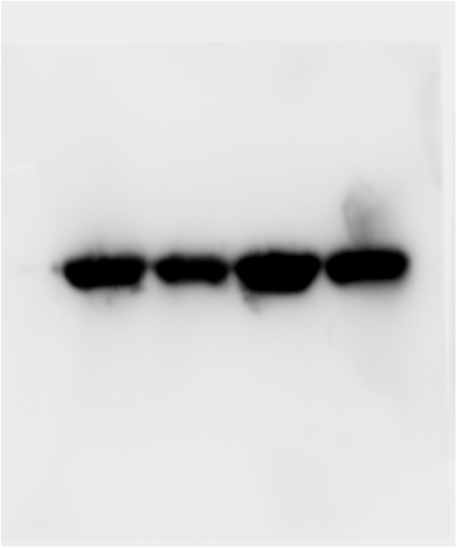

Supplement: Supplementary file 1 — Supplementary Information [file 41598_2017_18323_MOESM1_ESM.pdf]
